# Parental and Peer Support Matters: A Broad Umbrella of the Role of Perceived Social Support in the Association between Children’s Perceived Motor Competence and Physical Activity

**DOI:** 10.3390/ijerph18126646

**Published:** 2021-06-21

**Authors:** Cristina Menescardi, Isaac Estevan

**Affiliations:** 1 Activitat Física i Promoció de la Salut (AFIPS) Research Group, 46022 Valencia, Spain; cristina.menescardi@uv.es; 2 Department of Teaching of Musical Visual and Corporal Expression, University of Valencia, 46022 Valencia, Spain

**Keywords:** motor development, parents support, peer support, fundamental movement skills, self-perception, movement behavior

## Abstract

(1) Background: This study aimed to examine the role of social support in the relationship between perceived motor competence (MC) and physical activity (PA), according to the conceptual model of Motor Development. (2) Methods: Participants were 518 students (46.5% girls), 8–12 years old. By using a structural equation modeling approach, path analysis was used to test the actual-perceived MC relationship and the mediating influence of social support on the perceived MC–PA relationship. Analyses were done with age and sex as covariates. (3) Results: The results showed a good model fit (CFI = 0.98; RMSEA = 0.07; SRMR = 0.02), where actual MC was positively associated with perceived MC (ß = 0.26, *p* < 0.001), which in turn was positively related to social support (ß = 0.34, *p* < 0.001). The model showed the direct social support-PA path (ß = 0.42, *p* < 0.001) and the indirect path from perceived MC through social support to PA (ß = 0.14, *p* < 0.001). (4) Conclusions: This study confirms that social support mediates the perceived MC–PA relationship. As such, it is not only important to build and develop children’s actual and perceived MC, but also to promote social support for PA engagement.

## 1. Introduction

Physical activity (PA) participation during childhood is a behavioral key component for a healthy lifestyle, not only in the short but also long term [1]. However, few children meet the recommendations of 60 min of moderate to vigorous PA [2]. Primary school children (5–12 years old) do not seem to engage sufficiently in PA, which is seen as a current public health concern [3,4]. In fact, when children become adolescents, they take part in less PA [5,6]. As during childhood, individuals usually engage in increasingly complex PA (considered as motor tasks which include the combination of several fundamental motor skills (FMS) [7]) that requires appropriate levels of motor competence (MC) to participate; actual MC is a key aspect of children’s movement behavior and overall development [8]. Low levels of actual MC have been identified as a barrier to PA participation [9,10].

Actual MC is considered as the degree of proficiency in performing a wide range of FMS (e.g., jumping, catching, balancing), including the underlying mechanisms (e.g., coordination) required for daily life activities and for participation in more complex PA [7,11,12,13]. According to the conceptual model of Motor Development [7], an appropriate strategy to enhance children’s levels of PA is to empower not only actual but also perceived MC because it might foster motivation to PA participation and health trajectories [7,14], especially in those with low perceptions of MC [15], since they could self-report poorer performance than their actual competence warrants [16]. In this line, the existence of a dynamic and synergistic role between actual and perceived MC as contributors to PA involvement has been suggested, which can produce a positive or negative spiral impacting on children’s health [7,14].

Perceived MC refers to an individual’s awareness and belief in their capabilities in goal-directed human FMS and what would be considered as MC [14,17]. The role of perceived competence becomes apparent as children age and cognition develops [18]. Actually, those who perceived themselves as motor-competent tend to report high levels of PA [19,20]. As suggested above, perceived MC is important as a motivator for children’s PA participation [17] and has been also proposed as a mediator of the association between actual MC and PA [14], which has been consistent through object control [18,21] and locomotion skills [22]. In addition, recent systematic review and meta-analysis evidence showed that even though the association between actual and perceived MC seems to depend on intrapersonal factors such as sex, age, and the degree of alignment between the instruments used to capture actual and perceived MC, the results are inconclusive due to the lack and diversity of the studies, so that further research is required [23]. Future research could also seek to explore whether there are other confounding variables [23] that might affect the suggested association of actual MC, perceived MC, and PA [7], such as cultural or social factors [24].

The model proposed by Stodden et al. [7] shares some similarities with some recently proposed broader PA models [24] and physical literacy [25]. In these two other models, motor development is seen as not only influenced by individual-level factors (as in the model by Stodden et al. [12]), but also by social factors that are important to understand the progression from MC to health outcomes [25]. Social support is one of the social factors that could lead individuals to empower their motor development [10] and PA participation so that not only MC but also perceived social support may influence children’s PA [16]. Although self-perception is impacted by social support [26,27], individual-level models—which include factors that are important correlates of children’s health—often ignore the broader environmental context in which PA occurs [28]. For instance, a systematic review showed there is a positive association between family support with MC and PA in children and adolescents [11].

Social support is defined as a person’s perception of support and assistance from family, friends, and acquaintances [29] (p. 530). In childhood, social support can be seen as an isolated or conjunction of actions provided by significant others (e.g., parents and peers) that help an individual adopt or maintain a specific behavior [30]. Social support has been included in different theoretical frameworks (i.e., bio-ecological theory [26], social-cognitive theory [31], social-ecological theory [32], and ecological systems theory [16]), wherein it is postulated that multiple levels of factors such as physical and social environments can work together to influence children’s health behaviors. In terms of PA, the adoption or maintenance of active movement can occur mainly in three different ways of social support: (1) instrumental or direct support (e.g., sharing sports equipment, facilitating transport to local practices, and engaging in PA together), (2) psychological/emotional support (e.g., personal incentives, motivation, and encouragement for practices), and (3) informative support (e.g., acts of orientation, counseling, and talks about the importance and appropriate ways of engaging in PA). These three ways of support that children receive could be provided by parents and/or peers, influencing children’s levels of PA [16].

Given the role that parents play in children’s development and socialization, they have been identified as an especially important source of information, influencing and reporting in children’s PA and actual MC [33]. In addition to the household context, school and sports clubs are also considered social environments where peers can value individual perceptions. As children get older, they have strong social identity needs and tend to spend long periods of time with their friends rather than with family members [34], which could lead them to seek common contextual environments and activities such as PA [30]. Peer support may thus serve different functions such as companionship or practicing PA together, sharing equipment or transport, as well as providing esteem and empowering self-perception [35]. For instance, significant others can reduce the negative effects of children’s low self-perception [16] by encouraging them to remain persistent, giving them instruction and/or feedback, as well as allowing children to develop useful skills that build their perceived competence [36,37]. Despite their influence, little is known about to what extent social support is associated with perceived MC [16]. It is commonly hypothesized that the mechanisms of social support (instrumental, psychological, and informative support) provided by significant others can lead individuals to empower perceived competence and spend large periods of time-sharing common contextual environments, which in turn could influence the willingness to engage in PA [30]. As a result, higher levels of perceived social support provided by significant others entail higher levels of PA [30,34].

In fact, through observation and social interaction, children can reproduce the behaviors of their parents and peers in PA [16], but it is not clear to what extent social support can mediate the association between perceived MC and PA as a new aspect into the model of Motor Development [7]. The aim of the current study was thus to examine the role of social support (i.e., parental and peers) in the relationship between perceived MC and PA, according to the aforementioned model of Motor Development in primary school children. As social support has been found as a correlate of PA and can foster individual self-perception [35], our hypothesis is that perceived social support can mediate the association between children’s perceived MC and PA.

## 2. Materials and Methods

### 2.1. Participants

A non-probabilistic sampling method was used in six primary schools (public, private, and semi-private) in Valencia and its metropolitan area (Spain) that consented to participate. A total of 518 students (241 girls, 46.5%) from 8 to 12 years old (M = 9.64, SD = 1.08) participated voluntarily. Ethics approval was granted from the Ethics Committee of the first authors’ university (Reference Code 1259844), and parents or guardians provided written consent prior to the children’s participation.

### 2.2. Instruments

#### 2.2.1. Actual MC

The Canadian Agility Movement Skill Assessment (CAMSA) was used to assess actual MC [38]. This is a hybrid-oriented (process and product) motor test battery comprising seven tasks in a single circuit: (1) 2-footed jumping past 3 hoops; (2) sliding twice, one backward, over 3 m; (3) catching a ball; and then, (4) throwing the ball at a wall target 5 m away; (5) skipping for 5 m; (6) 1-foot hopping past six hoops; and (7) kicking a ball between 2 cones placed 5 m away. To create the CAMSA score (motor quotient), time taken to complete the obstacle course (time score; product-oriented criteria, converted from completion time into a 14-point score with higher values representing a shorter completion time on the CAMSA), and the quality of movement patterns assessed by 14 items (skill score; process-based criteria up to 14 points according to the skill performance criteria correctly executed) were used. The time to complete the circuit started when the participants began two-foot jumping and ended when they kicked the ball. The sum of both time and skill scores provided a CAMSA score range from 0 to 28 [38]. CAMSA score was calculated as the mean average of the two assessment trials. This assessment showed moderate-to-excellent inter- and intra-rater, and test–retest reliability in Canadian [38], Chinese [39], and Greek children [40]. Within this study sample, moderate-to-excellent test–retest intra-class correlation coefficients (ICCs) for time scores (ICC = 0.83, 95% CI (0.76, 0.88)), item scores (ICC = 0.70, 95% CI (0.58, 0.79)), and total CAMSA scores (ICC = 0.82, 95% CI (0.75, 0.87)) were found among 69 participants. Excellent intra-rater and inter-rater reliability for time scores (ICC = 0.99, 95% CI (0.98, 0.99) and ICC = 0.83, 95% CI (0.75, 0.89), respectively), item scores (ICC = 1.00), and total CAMSA scores (ICC = 0.99, 95% CI (0.99, 1.00) and ICC = 0.95, 95% CI (0.86, 0.96), respectively) were also found among 84 participants’ performances [41].

#### 2.2.2. Perceived MC

The pictorial instrument of Perceived Movement Skill Competence (PMSC) [42] was used. The PMSC assesses children’s perception of their MC in thirteen pictographic tasks (run, gallop, hop, jump, step, slide and skip, throw upper arm, catch, kick, hit, bounce, throw underarm, and racket). Children’s perception in each skill was rated from 1 (lower perception) to 4 (higher perception) as per protocol by using a double dichotomy process in a personal interview (8–10-year-old students) and supervised collective questionnaire session (11–12-year-old students) conducted by a research assistant following previous procedures [15,43,44], characterized by independent self-report without the possibility of peers’ influence as participants were not allowed to express their responses out loud. PMSC total score ranges between 13 and 52, with higher scores meaning a higher perceived MC [45]. The participants’ responses showed good reliability (α = 0.77).

#### 2.2.3. Social Support for PA

The Physical Activity Family and Friends Support Scales (PASS) by Norman et al. [46] adapted to the Spanish language were used [47]. This questionnaire, composed of 8 items, asked participants to rate from 1 (never) to 5 (every day) how often a member of their family or peers encourage them to do sports or PA during a typical week. The children’s responses showed good reliability (α = 0.72). Cronbach’s alpha was acceptable [48,49] for parental (α = 0.66) and peer (α = 0.52) support.

#### 2.2.4. Self-Reported PA

The Spanish version of the Physical Activity Questionnaire for Older Children (PAQ-C) [50] was used [51]. This is a self-reported 7-day recall questionnaire that assesses participation in different types of PA (e.g., walking, bicycling, running, swimming, dancing) as well as the level of PA during physical education (i.e., I was very active hardly ever, sometimes, quite often, or always) and type of PA during lunch break, recess (e.g., sat down, walked around, ran or played a little bit/quite a bit/most of the time), as well as the frequency in which they were very active after school, in the evenings, and at weekends last week (e.g., none; 1; 2 or 3; 4; 5 or more times) by the use of 9 items scored on a five-point Likert scale ranging from 1 (low PA level) to 5 (high PA level). The total score of PAQ-C was calculated as the mean of the scores for the 9 items [50]. The children’s responses showed good reliability (α = 0.72).

### 2.3. Procedures

Questionnaire administration (PMSC, PASS, and PAQ-C) were conducted prior to the actual MC assessment. CAMSA measurements were conducted in a large sports hall by two trained research assistants: one operated the camera while the other threw a ball to the participant/placed the ball on the kicking line for skills 3 (catch) and 7 (kick a ball). The children were allowed to perform two attempts and two assessment trials as fast as possible while performing the skills to the best of their ability according to the protocol [36]. Each child’s performance was video recorded by a 25 Hz Lumix TZ7 camera (Panasonic, Osaka, Japan ©) for subsequent coding.

### 2.4. Data Analyses

Descriptive and correlation analyses were calculated to examine the associations among study variables, age and sex using SPSS (Version 26; SPSS Inc., Chicago, IL, USA). A structural equation modeling (SEM) approach was used to test hypothesized direct relationships in model 1 (a) from actual MC to perceived MC, (b) from perceived MC to social support, and (c) to PA, as well as (d) from social support to PA; in addition to indirect relationships from perceived MC to PA mediated via social support through the model indirect procedure in Mplus v.8.s [52]. The amount of variability possibly explained by the selected factors was calculated with R-squared (R^2^) [22]. To further analyze parental and peer influence, model 2 was tested, similar to model 1, but divided social support into parental and peer support. Modification indices were analyzed for model respecification [53]. Both models 1 and 2 were computed with age and sex as covariates since both were correlated to the aforementioned variables in the models (see Table 1). Trivial (*r* < 0.20), low (*r* = 0.20–0.39), moderate (*r* = 0.40–0.59), moderately high (*r* = 0.60–0.79), and high (*r* > 0.80) consideration was used in the current study [54]. Cutoff values were used to establish the Goodness of fit at the Comparative Fit Index (CFI) > 0.90, Root Mean Square Error of Approximation (RMSEA), and Standardized Root Mean Residual (SRMR) < 0.08 to indicate good model fit [55].

## 3. Results

The means, standard deviations, and correlations among the study variables are presented in Table 1. The examination of the Pearson bivariate correlations revealed that the participants’ age and sex were associated with actual MC, perceived MC, social support, and PA. In other words, perceived MC, social support, and PA tend to decrease in older children while actual MC increases with their age. In addition, being a boy was associated with higher values in all the study variables than being a girl (sex: 0 = boys, 1 = girls).

**Figure 1:**
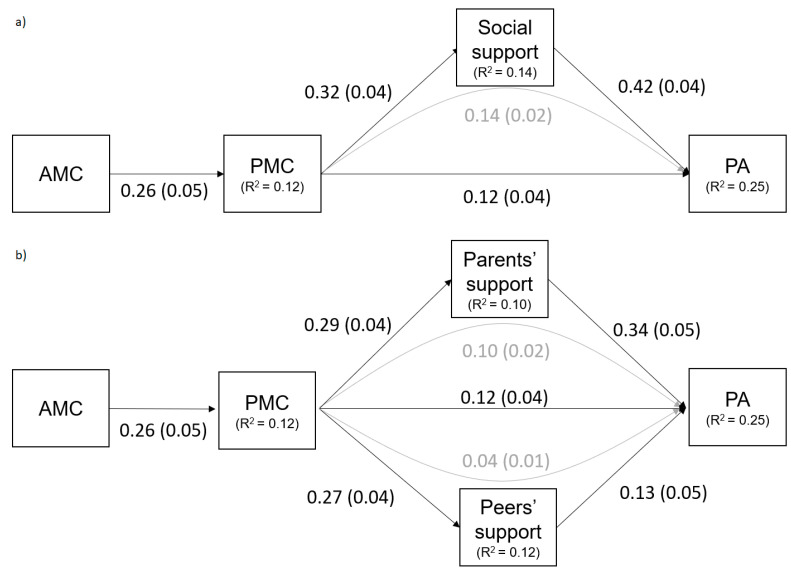
Path models for relations between actual motor competence (AMC), perceived motor competence (PMC), perceived Social Support, and physical activity (PA) (**a**), and between AMC, PMC, Parents’ and Peers’ Support, and PA (**b**). Models were represented with standardized coefficients (ß), standard errors (SE), and R-squares. (Note. Black and grey values refer to direct and indirect relationships, respectively. Solid arrows indicate significant relationships. Non-significant relationships were not found. Measured variables are represented with squares.).

Model 1 was computed with social support as a factor and fitted well with the data (χ^2^(2) = 5.068; CFI = 0.99; RMSEA = 0.05; SRMR = 0.02). Figure 1a represents the results of the path analysis, controlled by age and sex. In terms of direct paths, the model showed that the actual MC was positively associated with the perceived MC (ß = 0.26, *p* < 0.001), which in turn was positively related to social support (ß = 0.32, *p* < 0.001). A direct effect was found between social support for PA (ß = 0.42, *p* < 0.001) and between perceived MC and PA (ß = 0.12, *p* < 0.001), suggesting a mediation model with an indirect path from perceived MC to PA via social support (standardized indirect effect = 0.14; *p* < 0.001, 95% CI (0.09, 0.18)).

In model 2, social support was divided into two factors (i.e., family and peer support; Figure 1b) showing a good fit with the data (χ^2^(3) = 5.018; CFI = 0.99; RMSEA = 0.04; SRMR = 0.02) with the model being controlled by age and sex. According to model 1, the actual MC was positively associated with the perceived MC (ß = 0.26, *p* < 0.001), which in turn was also positively related to parental and peer support (ß = 0.29; ß = 0.27, *p* < 0.001, respectively). The association between parental and peer support was included (ß = 0.47) according to modification indices examination. In the model, both parental and peer support were related to PA (ß = 0.34; ß = 0.13, *p* < 0.001, respectively). Perceived MC was directly (ß = 0.12, *p* < 0.001) and indirectly associated with PA through parental (standardized indirect effect = 0.10; *p* < 0.001, 95% CI (0.06, 0.14)) and peer support (standardized indirect effect = 0.04; *p* = 0.001, 95% CI (0.01, 0.06)), suggesting a mediation model.

## 4. Discussion

Sufficient levels of PA participation in childhood contribute to healthy development and high MC, which in turn seem to be impacted by individual characteristics and the physical and social environment [11,56,57]. In the social environment, there are multiple reasons associated with the downward general trend in children’s PA (e.g., parental concerns, changing social norms around children’s independent mobility, an increase in sedentary leisure activities, and/or screen time, etc.), which limit children’s PA engagement [58]. It is thus important to recognize the underlying social mechanisms that explain children’s PA to understand their movement behavior and/or to prepare interventions focused on children’s health [15]. Based on the conceptual model of Motor Development [7], the purpose of the current study was, therefore, to examine the association of parental and peer support for PA with actual and perceived MC and PA engagement in primary school children. As hypothesized, social support was positively associated with children’s MC and PA.

The support of parents and peers can be seen as a correlate of the factors included in the conceptual model of Motor Development, such as MC and PA [11,24]. In the current study, social support was positively and moderately associated with children’s perceived MC and PA participation, which is in line with previous findings that found social support to be positively associated with levels of PA [16,30,34]. There was also a low positive association between social support and actual MC, so that, in line with Hulteen et al. [24], it seems that further cultural and/or social factors should be explored as a part of the aforementioned conceptual model [7,14] since the inclusion of other psychological elements such as perceived social support can be important to understand the progression from MC to health outcomes [25]. Consistent with prior research showing that children’s PA is impacted by social support [30], our study extended these results to the emerging motor development field and found that the more support children received from their parents, the more they engaged in PA and the higher their perceived and actual MC.

Having adequate MC assists in promoting enjoyment and engagement in a diversity of physical activities and contributes to positive trajectories of health (i.e., physical, mental, and socio-emotional) at any level (e.g., high performance and/or recreational level) [59]. The conceptual model of Motor Development [7,14] establishes, among other factors, straightforward bidirectional pathways as a result of time like that between the actual and perceived MC with PA. However, a recent systematic review showed research in motor development did not find consistent evidence supporting the postulated direct pathway because studies in the field used heterogeneous methods (e.g., level of alignment between actual and perceived MC assessments, assessing different dimensions of MC, using self-reported vs. objective instruments to assess PA, etc.) and sample characteristics such as age, sex, or developmental status [23]. First, regarding the association between actual and perceived MC, revised studies analyzing 7–12-year-old Belgian [60], Chinese [61], Iranian [62] and Italian children [63], in which diverse domains of perceived MC (i.e., perceived physical competence and/or perceived sport/athletic/MC) were measured reported small pooled effects [23]. Our results using partially-aligned assessments, support the aforementioned postulations in terms of a trivial-low association between these two factors in middle childhood. Secondly, regarding the association between actual MC and PA, a trivial association was also found. Even though in middle childhood MC seems to drive engagement in PA [7], our results were consistent with those of Barnett et al. [11], who found an indeterminate association mainly due to the diversity of the methodological approaches, different assessments, and individual characteristics (e.g., children’s sex and age), which limit the interpretation of this association [11,64]. Thirdly, considering the association between perceived MC and PA, children’s self-perception was also found to be a correlate of PA engagement [65,66]. It seems that, in middle childhood, higher self-perception in motor skill competence entails greater PA engagement, which is corroborated by our results, which found a low association between these two factors. A recent US study on Hispanic 10–11-year-old children [67] showed a low association between PA and perceived MC in object control skills. Interestingly, the authors of this study did not operationalize perceived MC for analysis, as we did in the current study. Whereas Zhang et al. [67] studied the relationship of each measured object control skill separately with self-reported PA, we examined the association between self-reported PA and perceived MC, integrating locomotive and object control skills into the whole construct. In any case, it is promising that perceived MC can contribute to PA engagement [60,67]. Likewise, because of the low levels of association, the relationship of actual MC with perceived MC and PA does not seem as straightforward as postulated in conceptual models.

One novelty of the current study was the incorporation of social support into theoretical frameworks in children’s motor development. Our purpose was to examine the mediation role of social support (i.e., parental and peers) in the relationship between perceived MC and PA in primary school children. In this regard, the findings showed that higher levels of perceived MC accompanied by higher levels of perceived social support led to higher levels of PA engagement. In agreement with our hypothesis, our findings exhibited the mediation effect of social support on the association between children’s perceived MC and PA. In the current study, we extend the previous literature in motor development [7,14,24] by exploring underlying mechanisms through which healthy lifestyles can be established, i.e., actual MC was positively associated with perceived MC, which in turn was related to PA engagement through social support, confirming the positive association between social support (especially family) and PA in children [11]. Children’s perception of interpersonal relationships with significant others, or what is considered as the ways in which parents and friends treat the child by what they say and what they do in that setting [68], seems to impact their healthy lifestyles. In addition, it is not only the behavioral setting that explains children’s behavior but the individual’s perception and interpretation of this setting in both time and space. To understand children’s motor development, scientists should therefore not only focus on researching the children’s environment objectively but also on how children see it [69].

We also explored the mediation role of perceived social support according to the source of information, i.e., by parents and peers. In the current study, parental and peer support mediated the association between perceived MC and PA engagement. Whereas parental support was moderately related to PA engagement, perceived peer support was loosely associated with PA. During childhood, parental support seems to impact motor development and PA, which is in agreement with our findings involving 8–12-year-old children, whereas as children transit into adolescence, the impact of peer support gains relevance [30,58], i.e., parents seem to be the primary socializing agents for children’s PA engagement formation throughout childhood and thus, represent a key factor to target [70]. A recent systematic review has shown that parents’ support is an effective correlate for PA engagement in terms of co-participation, family involvement, emotional, informational, companionship, and instrumental [58], which supports the notions of the bioecological theory [26]. Bronfrenbrenner [26,68] postulated the existence of different systems to understand human development by interrelated ecological levels (i.e., microsystem, mesosystem, exosystem, and macrosystem). The microsystem was described as a setting within which the individual is behaving at a given moment in their life; that is, human development involves the complex relations between the developing individual and the context in an immediate setting containing the person [68]. Among others, the microsystem encompasses social roles and interpersonal relations experienced by the individual in a particular social setting that foster or limit engagement in activities. So, taking into account that identifying correlates of health behavior seems to be critical for developing and refining successful behavior change interventions for health [27,58], the current study provided insight by highlighting the importance of social support as a correlate of children’s PA engagement and, consequently, motor development. Thus, in line with Hulteen et al. [24], it seems relevant to start incorporating new psychological aspects to help researchers, teachers, and stakeholders to understand the association of the factors in children’s motor development with their health. In this line and in agreement with Zhang et al. [67], understanding the roles of actual and perceived MC as well as significant others’ support in predicting children’s health-related outcomes, such as PA, can provide meaningfully practical implications for future interventions in environments such as in- and/or out-of-school settings aimed at promoting PA and eliminating health disparities.

As far as the authors know, few studies have examined the pathways of the conceptual model of motor development in the same study (e.g., [71]). This is one of the first studies within the sample studied (Spanish children) that incorporates social support as a mediator of the association between perceived MC and PA engagement. Nonetheless, the study is not absent of limitations: (1) the cross-sectional design in conjunction with the fact of not being a probabilistic sampling may inflate the hypothetical evidence of the postulated associations in the conceptual model; (2) the instruments used include: (a) the use of self-reported questionnaires, which can over-report PA levels, (b) the absence of full alignment between AMC and PMC assessment, and (c) limited reliability in parental and peers’ social support, which even though could be acceptable [48,49], it can also be seen as poor [72]. Another limitation was the absence of variables related to Physical Fitness (e.g., cardio-respiratory), which have also been suggested as possible mediators of the AMC–PA relationship. To confirm the appropriateness of the aforementioned dynamic pathways of the model, future longitudinal and causal relationships should be conducted by prospective and randomized trials, respectively, as well as more reliable PA measurements by using accelerometers and the Physical Fitness variables to further describe factors that are related to children’s PA engagement.

## 5. Conclusions

The current study takes a significant step toward uncovering the role of social support (parental and peer support) as a mediator of the relationship between children’s perceived MC and PA. The current findings are consistent with the hypothesis that parents’ and peers’ encouragement and provision of opportunities and experiences for children’s engagement in PA lead to higher levels of children’s PA. For this reason, social agents such as parents, in collaboration with teachers, should build strategies to develop children’s actual and perceived MC as well as to promote students’ support for PA engagement in and out of school to foster healthy lifestyle behaviors in primary school children.

## Figures and Tables

**Table 1:** Descriptive statistics and correlations between study variables.

		Correlations						
	*M (SD)*	1	2	3	4	5	5a	5b
1. Sex ^1^	-	-						
2. Age	-	−0.04						
3. Physical Activity	3.23 (1.07)	−0.15 **	−0.10 *					
4. Perceived MC ^2^	40.02 (5.83)	−0.18 **	−0.18 **	0.29 **				
5. Social Support	3.21 (0.83)	−0.10 *	−0.15 **	0.48 **	0.37 **			
5a. Parents Support	3.00 (1.03)	−0.10 *	−0.11 *	0.46 **	0.31 **	0.88 **		
5b. Peers Support	3.42 (0.90)	−0.06	−0.16 **	0.36 **	0.33 **	0.84 **	0.49 **	
6. Actual MC	19.48 (3.40)	−0.21 **	0.38 **	0.12 *	0.18 **	0.12 *	0.10 *	0.11 *

^1^ Boys = 0; girls = 1; ^2^ MC refers to motor competence. * *p* ≤ 0.05; ** *p* ≤ 0.01.

## Data Availability

The data will be available on request.
